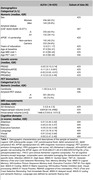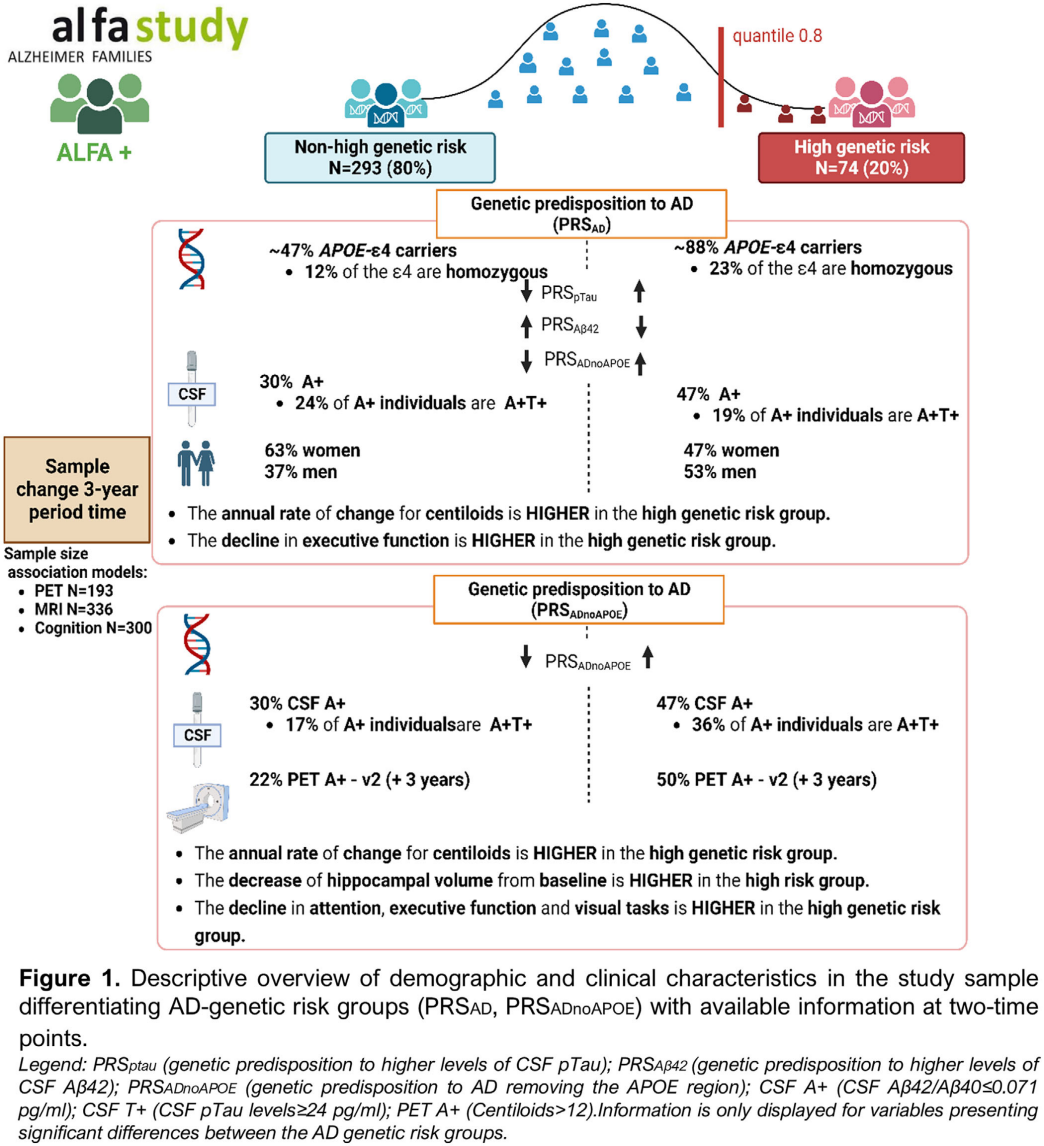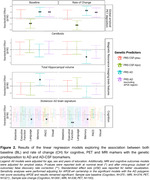# Genetic predisposition to clinical AD and AD pathology is differentially associated with baseline and 3‐year period change for specific AD‐related markers

**DOI:** 10.1002/alz.093688

**Published:** 2025-01-09

**Authors:** Patricia Genius, Blanca Rodríguez‐Fernández, Mahnaz Shekari, Gonzalo Sánchez‐Benavides, David López‐Martos, Carolina Minguillon, Manel Esteller, Arcadi Navarro, Juan Domingo Gispert, Natalia Vilor‐Tejedor

**Affiliations:** ^1^ Barcelona?eta Brain Research Center (BBRC), Pasqual Maragall Foundation, Barcelona Spain; ^2^ 30, Carrer de Wellington, Barcelona Spain; ^3^ Universitat Pompeu Fabra, Barcelona Spain; ^4^ Centro de Investigación Biomédica en Red de Fragilidad y Envejecimiento Saludable (CIBERFES), Instituto de Salud Carlos III, Madrid Spain; ^5^ IMIM (Hospital del Mar Medical Research Institute), Barcelona Spain; ^6^ Cancer Epigenetics and Biology Program, Bellvitge Biomedical Research Institute, L'Hospitalet (Barcelona) Spain; ^7^ Centre for Genomic Regulation (CRG), Barcelona Institute of Science and Technology (BIST), Barcelona Spain; ^8^ Centro de Investigación Biomédica en Red Bioingeniería, Biomateriales y Nanomedicina, Instituto de Salud Carlos III, Madrid Spain; ^9^ Barcelona?eta Brain Research Center (BBRC), Barcelona Spain

## Abstract

**Background:**

Alzheimer’s disease (AD) is a complex neurodegenerative disorder characterized by early changes in brain structure and cognitive function before the age of onset. This study investigated whether the genetic load for clinical AD and AD pathology predicts AD‐related brain and cognitive changes over a 3‐year period, targeting the preclinical phase in cognitively unimpaired (CU) middle‐aged individuals.

**Method:**

The sample of the study was defined by 429 CU middle‐aged participants at risk of AD from the ALFA+ nested cohort with available information on genetics, brain imaging markers and cognitive data [Table 1]. A subset of them had information at two‐time points (N = 367) [Figure 1]. Genetic predisposition for clinical AD (PRSAD and PRSADnoAPOE) and AD pathology (PRSAβ42‐CSF and PRSpTau‐CSF) were calculated. We used linear regression models to assess the association between each PRS and global amyloid PET deposition expressed in Centiloids, AD Dickerson MRI‐signature, hippocampal volume and cognitive outcomes, at baseline and rate of change over visits after controlling for confounders. PET and MRI outcomes were defined as the annual rate of change. Cognitive outcomes were z‐scores of the two‐time points difference. Models were adjusted for time between visits.

**Result:**

At baseline, higher genetic predisposition to clinical AD was associated with increased amyloid‐PET levels and higher average thickness of AD‐affected regions. Additionally, higher PRSAD was associated with reduced AD‐related cognitive performance and worse language [Figure 2]. Over the three‐year period, AD‐genetic load predicted higher annual amyloid accumulation in the brain, independently of APOE. Conversely, genetic predisposition to higher CSF Aβ42 levels predicted lower annual rate of change for Centiloids. PRSAD was also associated with annual reduction of hippocampal volume. Finally, genetic predisposition to AD beyond the APOE region was associated with executive functioning rate of change[Figure 2].

**Conclusion:**

The results of this study showed the role of genetic predisposition to AD, affecting amyloid PET deposition, brain structure changes, and cognitive decline. These findings emphasized the importance of considering a comprehensive range of genetic markers in risk assessments for AD, particularly in its early stages, and highlight the potential of personalized genetic approaches to enhance predictions and interventions in the management of AD.